# Preparation, Characterization and Formation Mechanism of High Pressure-Induced Whey Protein Isolate/κ-Carrageenan Composite Emulsion Gel Loaded with Curcumin

**DOI:** 10.3390/gels10080542

**Published:** 2024-08-21

**Authors:** Xiaoye He, Shuang Ren, Hu Li, Di Han, Tianxin Liu, Meishan Wu, Jing Wang

**Affiliations:** 1 Institute of Food and Nutrition Development, Ministry of Agriculture and Rural Affairs, Beijing 100081, China; 2 The Key Laboratory of Food Resources Monitoring and Nutrition Evaluation, Ministry of Agriculture and Rural Affairs, Beijing 100081, China; 3 Department of Food Science, University of Tennessee, Knoxville, TN 37996, USA

**Keywords:** high pressure processing, curcumin, emulsion gel, structure, formation mechanism

## Abstract

In order to explore the formation mechanism of the emulsion gel induced by high pressure processing (HPP) and its encapsulation and protection of functional ingredients, a curcumin-loaded whey protein isolate (WPI)/κ-carrageenan (κ-CG) composite emulsion gel induced by HPP was prepared. The effect of pressure (400, 500 and 600 MPa), holding time (10, 20 and 30 min) and concentration of κ-CG (0.8%, 1.0% and 1.2%, *w*/*v*) on the swelling rate, gel strength, the stability of curcumin in the emulsion gel, water distribution and its mobility, as well as the contents of interface protein were characterized. The results showed that the addition of κ-CG significantly reduced the protein concentration required for the formation of emulsion gel induced by HPP and greatly reduced the swelling rate of the emulsion gel. The gel strength and storage stability of the composite emulsion gels increased with the increase in pressure (400–600 MPa) and holding time (10–30 min). When the pressure increased to 500 MPa, the stability of curcumin in the emulsion gel significantly improved. When the ratio of WPI to κ-CG was 12:1 (the κ-CG concentration was 1.0%), both the photochemical and thermal stability of curcumin were higher than those of the other two ratios. The HPP significantly increased the mobility of monolayer water in the system, while the mobility of multilayer water and immobilized water was significantly reduced. Increasing the holding time and the concentration of κ-CG both can result in an increase in the interfacial protein content in the oil/water system, and the HPP treatment had a significant effect on the composition of the interfacial protein of the emulsion gel.

## 1. Introduction

Emulsion gel has excellent protection and slow-release effects for lipophilic bioactive agents by incorporating, stabilizing and delivering them, and it is often used in sausage, yogurt, cheese and dairy products, which are often prepared using food-grade proteins and/or polysaccharides that form a three-dimensional polymer or particle network in water [1]. In recent years, high incidences of chronic diseases (such as diabetes, hypertension and cancer) in both developed and developing nations has aroused widespread concern regarding the enhancement of food functionality and nutrition through the inclusion of enriched bioactive ingredients [2,3]. Therefore, the utilization of emulsion gel has become a research hotspot in the field of food-grade delivery systems. In addition, emulsion gels are also commonly used in the development of novel foods. By regulating the interaction between the filling oil phase and the gel matrix, thereby adjusting the lubrication properties of the food product, emulsion gel can be developed to help relieve the pain of patients with chewing and swallowing disorders. Additionally, the swelling sensation caused by the gel matrix can be used to simulate the taste of fat and develop new types of lipid-lowering food [4].

Although some non-thermal induction methods such as acid induction, enzyme induction and ion induction have been widely used to encapsulate thermally unstable functional ingredients, these methods more or less introduce impurities and affect the sensory properties and texture of the product, thereby limiting the application of gels in certain food systems. In comparison, high pressure processing (HPP) has broad application prospects in the preparation of emulsion gels because of its advantages, such as high efficiency, low consumption, no introduction of impurities and preservation of original flavor [5]. Previous studies have shown that HPP can significantly change the structure and intermolecular interactions of biomacromolecule polymers. For example, under sufficient pressure, the globular protein dispersed in the aqueous solution will unfold and aggregate to form a network structure through hydrophobic interaction and disulfide bonding, which is conducive to the formation of emulsion gels [6].

Because of its nutritional value, emulsification and gel properties, whey protein isolate (WPI) is widely used as a wall material for delivery systems such as emulsions, gels, microcapsules and microgel particles, and to encapsulate unstable bioactive agents such as β-carotene, curcumin and lutein [7]. The κ-carrageenan (κ-CG) has a high proportion of anionic sulfuric acid groups and can form complexes with WPI, and it can improve the gel structure by filling the protein network through hydrogen bonding [8]. The presence of polysaccharides can promote the interactions between protein molecules that are unfolded under high pressure [9]. Li et al. showed that the combination of κ-carrageenan and β-lactoglobulin can significantly reduce the protein concentration required for gel formation, which is mainly because carrageenan and protein can form a coupled network structure to enhance the strength of the gel [10]. 

Although there have been a few studies about high pressure-induced food gel, most of them are more focused on meat products, being less involved in the encapsulation of bioactive ingredients. In addition, there is still limited research data with respect to the formation mechanism of high pressure-induced emulsion gels. Therefore, this article aims to prepare WPI and WPI-CG emulsion gels by HPP induction and study the effect of pressure, holding time and concentration of κ-CG on the swelling rate, mechanical properties and stability of curcumin-loaded WPI-CG composite emulsion gel induced by HPP. Moreover, the formation mechanism of the emulsion gels was investigated by low-field nuclear magnetic resonance (LF-NMR), the measurement of interface protein content and gel electrophoresis for the first time. These results can provide a theoretical basis for the application of HPP in the preparation of food gels and the encapsulation of bioactive ingredients.

## 2. Results and Discussion

### 2.1. Swelling Rate

**Figure 1:**
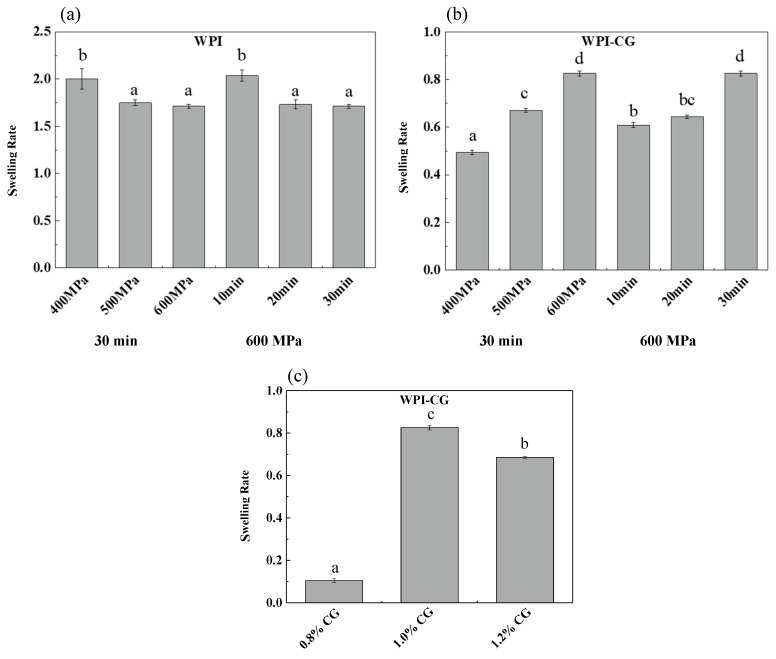
Swelling rate of emulsion gels with different treatment pressures, holding time (**a**,**b**), and κ-CG concentrations (**c**). Note: In (**a**,**b**), the whey protein isolate (WPI) concentration is 12% (*w*/*v*) and the whey protein isolate/κ-carrageenan (WPI-CG) ratio is 12:1. In (**c**), the pressure is 600 MPa, and the holding time is 30 min. Different letters in the same figure indicate a significant difference (*p* < 0.05, n = 3).

The swelling capacity of gels determines the degree of water reabsorption, which highly depends on the porosity and water absorption capacity of the gelling agents [11]. Swelling plays an important role in the mechanical properties, stability, digestion of gels and subsequent release of delivered bioactive components [12]. The effect of pressure, holding time and concentration of κ-CG on the swelling rate of two different emulsion gels is shown in Figure 1. The swelling time of the WPI emulsion gel was 2 h, and the swelling time of the WPI-CG emulsion gel was 12 h. It can be seen that the swelling rate of the WPI emulsion gel was significantly greater than that of the WPI-CG emulsion gel. The reason might be that the addition of κ-CG made the gel stronger and reduced the diffusion rate of water molecules in the gel. For the WPI emulsion gel, as the pressure increased from 400 MPa to 500 MPa, and the holding time increased from 10 min to 20 min, the swelling rate decreased, which caused the gel network structure to become firm, and the gel hardness increased. However, after the pressure and holding time increased to 600 MPa and 30 min, respectively, the swelling rate did not change significantly, indicating that after being treated at 500 MPa for 20 min, the WPI emulsion gel formed a relatively stable network structure, and increasing the treatment pressure and time had no significant effect on the gel structure. For the WPI-CG emulsion gel, as the pressure and holding time increased, the swelling rate increased, which is speculated to be related to the texture and water loss of the WPI-CG emulsion gel. As the treatment pressure and holding time increase, the emulsion gel loses more water and therefore, more space is left for the external water molecules.

It can be seen from Figure 1c that the swelling rate of the gel increased first and then decreased with the increase in the κ-CG concentration. As the concentration of κ-CG increased, more water molecules were lost from the gel after HPP treatment, providing more space for external water molecules. However, at the same time, the hardness of the gel increased, which made it more difficult for water molecules to enter the gel. Therefore, the swelling rate first increased and then decreased.

### 2.2. Texture Profile Analysis (TPA)

**Figure 2:**
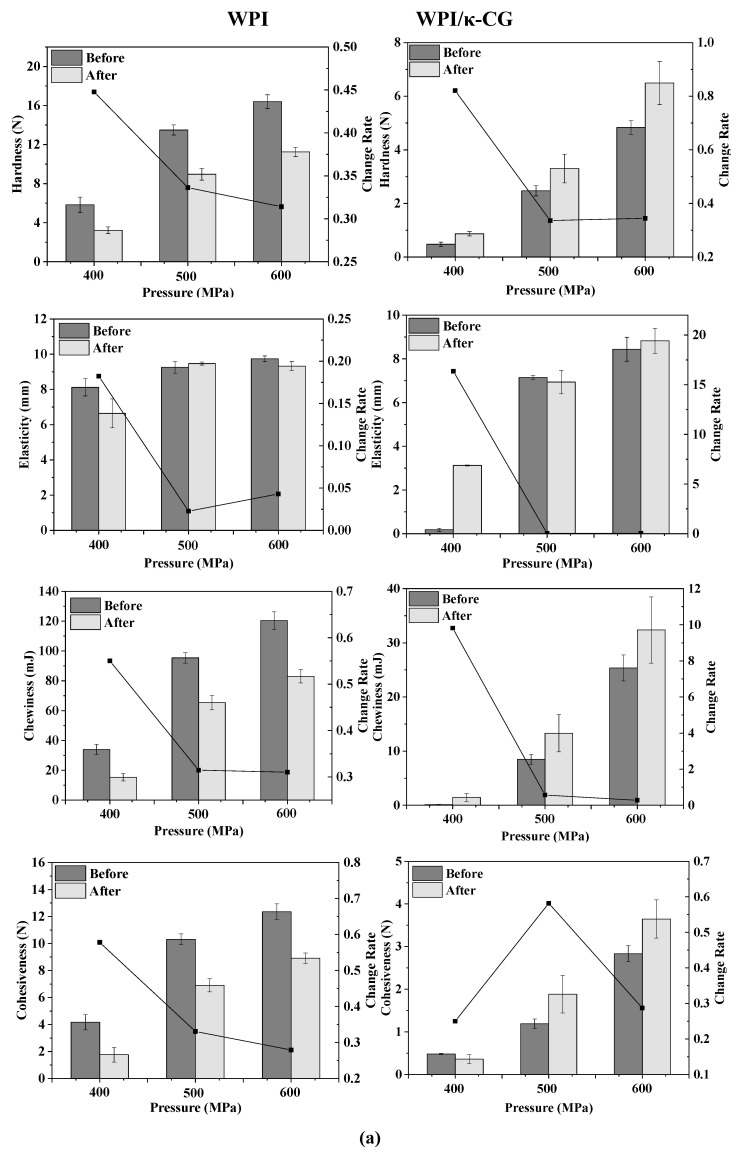

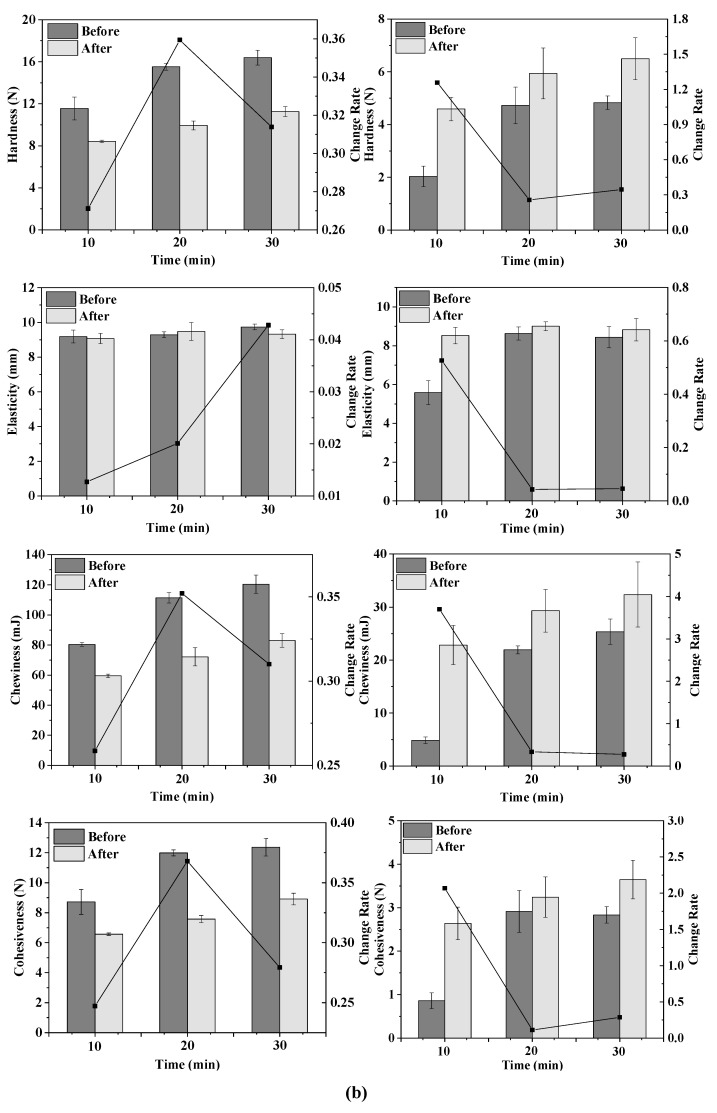

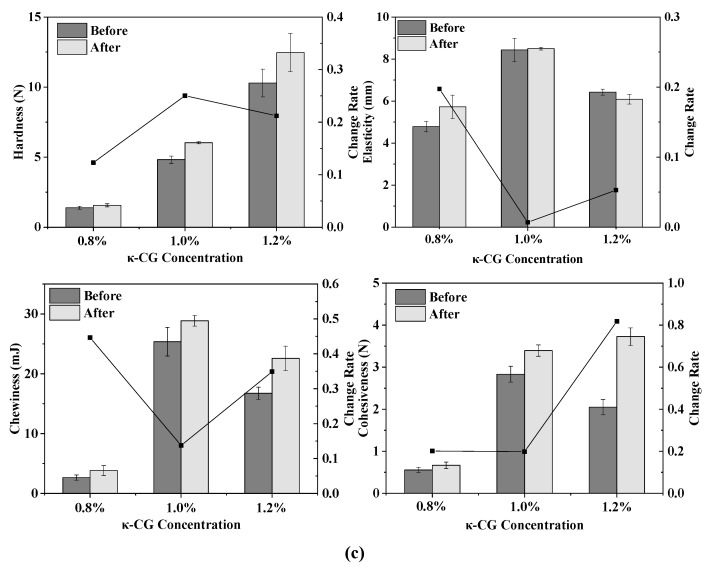
Texture change trends of emulsion gels with different treatment pressures (**a**), holding times (**b**) and κ-CG concentrations (**c**) before and after storage for 15 days. Note: in (**a**,**b**), the whey protein isolate (WPI) concentration is 12% (*w*/*v*) and the whey protein isolate/κ-carrageenan (WPI-CG) ratio is 12:1. In (**a**), the holding time is 30 min, and in (**b**), the pressure is 600 MPa. In (**c**), the pressure is 600 MPa, and the holding time is 30 min.

The texture change trend of emulsion gels treated by different pressures before and after stored for 15 days is shown in Figure 2a. As the pressure increased, the TPA parameters (hardness, elasticity, cohesiveness and chewiness) of all samples increased, indicating that the gel strength was enhanced. Wei et al. demonstrated that with the increase in pressure, the content of disulfide bonds in the WPI protein gel increased, the cross-linking between the protein molecules was enhanced and the gel network structure formed was denser [13]. For the WPI emulsion gel, the hardness, chewiness and cohesiveness of the gel were reduced after storage, but the elasticity did not change significantly. For the WPI-CG emulsion gel, the change in TPA parameters after storage was exactly the opposite of that observed in the WPI emulsion gel. The hardness, elasticity, chewiness and adhesiveness of the samples after storage all increased. This could be due to the relatively poor water-holding capacity of the WPI-CG emulsion gel. The partial loss of water during storage caused the strength of the gel to increase.

As shown in Figure 2b, the TPA parameters of the emulsion gels increased with the increase in the treatment time, indicating that the stability of the emulsion gels was improved. For the WPI emulsion gel, as the holding time increased, the hardness, chewiness, and cohesiveness increased, and the change rate after and before storage first increased and then decreased. For the WPI-CG emulsion gel, the change rate of TPA parameters of the sample treated at 600 MPa for 10 min after and before storage was relatively large. It could be due to the fact that the treatment time was too short, resulting in an unstable gel structure. There was no significant difference in the texture of the emulsion gel treated for 20 min and 30 min, indicating that after the treatment time reached a certain level, the gel network structure was fully formed. Therefore, further increasing the treatment time will no longer significantly increase the strength of the gel network.

It can be seen from Figure 2c that with the increase in the κ-CG concentration, the elasticity, chewiness and cohesiveness of the samples before storage all increased first and then decreased. On the whole, the WPI-CG emulsion gel with 1.0% κ-CG had better storage stability, indicating that there was an optimal concentration ratio of WPI to κ-CG. Research by Li et al. showed that the compound of polysaccharides and proteins can significantly reduce the protein concentration induced by HPP to form gels [10]. The existence of the synergistic interaction between β-lactoglobulin and carrageenan can form a coupled network structure to enhance the strength of the gel. However, the proportion of polysaccharide should be controlled within a reasonable range. A polysaccharide concentration that is too low will lead to insufficient cross-linking between the polysaccharide and protein molecules, resulting in a loose gel structure. A polysaccharide concentration that is too high will lead to excess polysaccharide and affect the coupling between the protein molecules and the polysaccharide. In this experiment, when the concentration ratio of WPI to κ-CG in the gel was 12:1 (κ-CG concentration is 1.0%), the WPI-CG emulsion gel induced by HPP exhibited better stability.

### 2.3. Stability of Curcumin

#### 2.3.1. Photochemical Stability

**Figure 3:**
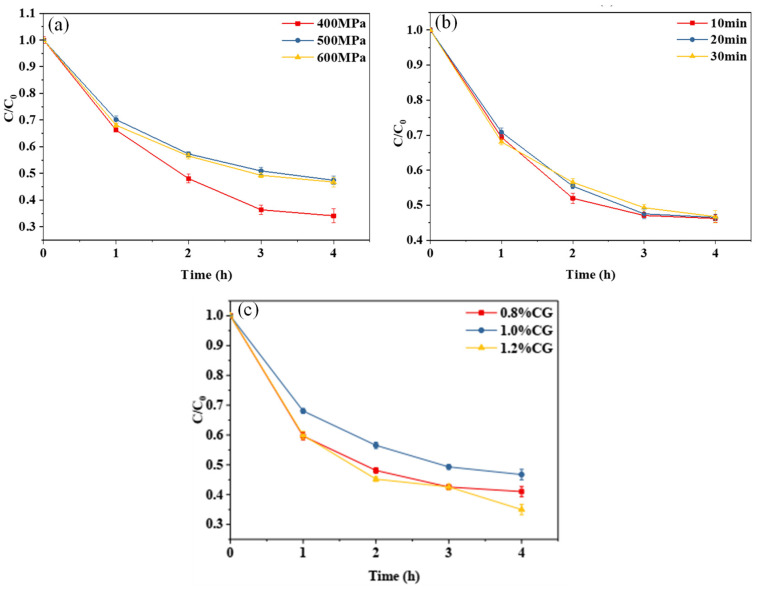
Photodegradation trend of curcumin in whey protein isolate/κ-carrageenan (WPI-CG) emulsion gel with different treatment pressures (**a**), holding time (**b**) and κ-CG concentrations (**c**). Note: all samples were exposed to light conditions (35 °C, 0.35 W/m^2^) for 4 h. In (**a**,**b**), the WPI concentration is 12% (*w*/*v*) and the WPI-CG ratio is 12:1. In (**a**), the holding time is 30 min, and in (**b**), the pressure is 600 MPa. In (**c**), the pressure is 600 MPa, and the holding time is 30 min.

Figure 3a,b show the curves of the retention rate of curcumin embedded in the WPI-CG emulsion gel with different treatment pressures and holding times as a function of light exposure time. The photochemical stability of the gel treated at 400 MPa was significantly lower than that of the other samples. When the pressure was increased to 500 MPa, the photochemical stability of the curcumin no longer increased with further increases in pressure. This indicates that increasing the treatment pressure within a certain range can improve the photostability of curcumin in the WPI-CG emulsion gel. This is because, as the treatment pressure increases, more disulfide and hydrogen bonds are formed between protein molecules, making the gel structure more compact, and curcumin can be better embedded in the gel network structure. It could be seen from the curve that different holding times had no significant effect on the photochemical stability of curcumin embedded in the composite gel. It can be seen from Figure 3c that the concentration of κ-CG had a significant effect on the stability of curcumin. In the κ-CG concentration range of 0.8% to 1.2%, the curcumin embedded in the emulsion gel with 1.0% κ-CG showed better photochemical stability. An appropriate protein–polysaccharide ratio can improve the strength of the emulsion gel [10], thereby improving the photostability of curcumin. In this experiment, when the concentration ratio of WPI to κ-CG was 12:1 (κ-CG concentration is 1.0%), the curcumin embedded in the emulsion gel had the best photochemical stability.

#### 2.3.2. Thermal Stability

**Figure 4:**
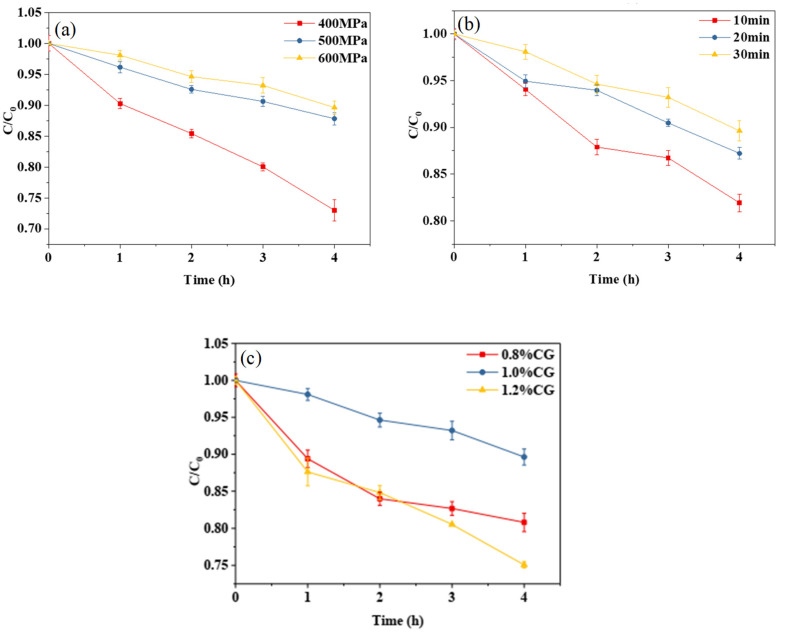
Thermal degradation trend of curcumin in whey protein isolate/κ-carrageenan (WPI-CG) emulsion gel with different treatment pressures (**a**), holding time (**b**) and κ-CG concentrations (**c**). Note: in (**a**,**b**), the WPI concentration is 12% (*w*/*v*) and the WPI-CG ratio is 12:1. In (**a**), the holding time is 30 min, and in (**b**), the pressure is 600 MPa. In (**c**), the pressure is 600 MPa, and the holding time is 30 min.

It can be seen from Figure 4a,b that, with the increase in treatment pressure or holding time, the degradation rate of curcumin decreased, indicating that, within a certain range, the higher the treatment pressure or the longer the holding time, the better the thermal stability of the curcumin embedded in the composite emulsion gel becomes. It can be seen from Figure 4c that the κ-CG concentration had a significant effect on the thermal stability of the curcumin embedded in the WPI-CG emulsion gel. With the concentration ranging from 0.8% to 1.2% (*w*/*v*), the degradation rate of curcumin under 80 °C heating conditions exhibited a first decrease and then an increase. The thermal stability of curcumin tended to reach the maximum when the κ-CG concentration was 1.0%. The conclusion and reasoning are consistent with those of the photochemical stability analysis.

### 2.4. LF-NMR

**Figure 5:**
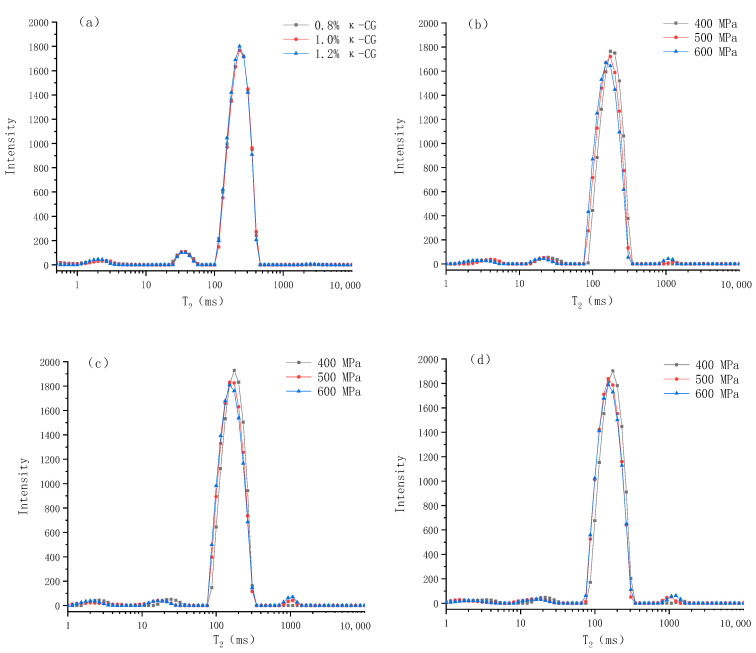
Changes in relaxation time (T_2_) of whey protein isolate/κ-carrageenan (WPI-CG) emulsion gels under different conditions: (**a**) under normal pressure; (**b**) 0.8% κ-CG; (**c**) 1.0% κ-CG; (**d**) 1.2% κ-CG. Note: in Figure 5, the holding time is 30 min.

LF-NMR is a widely used method to determine water distribution and its mobility. Compared with traditional methods, LF-NMR is a non-destructive testing method, which will not damage the micro and macro structure of the gel [14]. The alterations in the transverse relaxation time (T_2_) of the protons in the gel and the corresponding exponential populations (P) can be used to quantify the changes in the water distribution and mobility caused by the experimental factors [15]. According to previous literature, researchers can usually observe 3–4 signal peaks, but the T_2_ (ms) corresponding to each peak position is often different, which can be attributed to the differences in equipment, NMR parameters and raw materials [16]. As can be seen from Figure 5, there are four peaks, centered on 0~5 ms (T_21_), 10~50 ms (T_22_), 100~500 ms (T_23_) and 1000~1500 ms (T_24_), representing monolayer water that is strongly hydrogen-bonded with polar groups of large molecules, multilayer water that is weakly hydrogen-bonded with large molecules, immobilized water that is confined in the microscopic network structure of gel and accounts for about 90% of the total water, and free water, outside the microscopic network structure of the gel, respectively [17].

Table 1 shows the specific values and significant differences of the LF-NMR spin–spin relaxation time (T_2_) and the corresponding exponential populations (P) of emulsion gels under different pressures and κ-CG concentrations. It can be seen that after the emulsion gel was formed through HPP, T_21_ was significantly increased, T_22_ and T_23_ were significantly decreased, and with the increase in pressure, T_22_ and T_23_ decreased continually. This indicates that after the emulsion gel was formed, the mobility of the monolayer water in the system was significantly increased, while the mobility of the multilayer water and immobilized water were significantly decreased. With the increase in pressure, the mobility of the latter two were continuously decreased. In addition, HPP significantly reduced P_21_ and P_22_, and increased P_23_ and P_24_, which suggests that HPP significantly reduced the content of monolayer water and multilayer water in the system and converted them into immobilized water and free water. It can be seen from the table that increasing the concentration of κ-CG had no significant effect on the relaxation time (*p* > 0.05), indicating that κ-CG had no significant effect on the mobility of water molecules in the emulsion gel. However, the increase in κ-CG significantly reduced P_23_, increased P_21_ and P_22_, indicating that κ-CG changed the relative content of different kinds of water in the system, increased the ratio of monolayer water and multilayer water, and reduced the ratio of immobilized water, thereby increasing the water holding capacity of the emulsion gel.

### 2.5. The Content of Interface Protein

**Figure 6:**
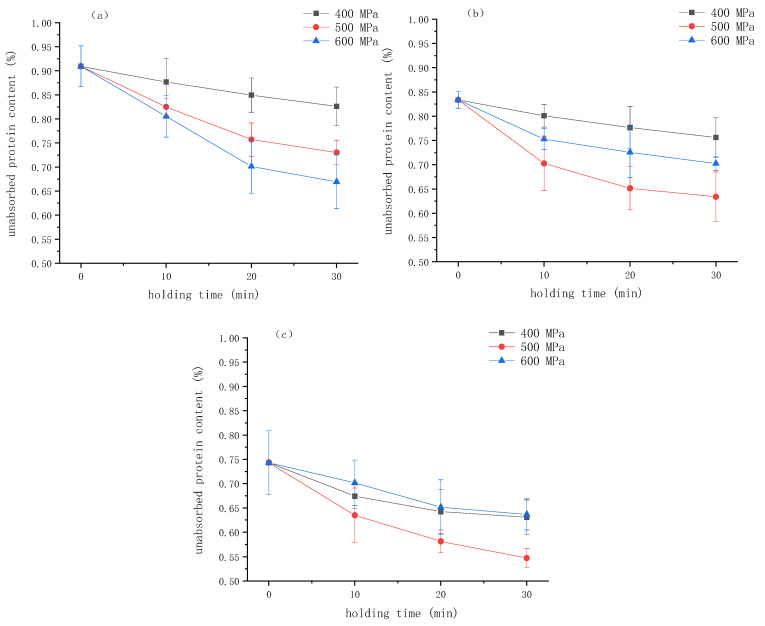
The percentage of unabsorbed protein in emulsion gels under different pressure and κ-carrageenan (κ-CG) concentrations versus time: (**a**) 0.8% κ-CG, (**b**) 1.0% κ-CG, (**c**) 1.2% κ-CG. Note: in Figure 6, the holding time is 30 min.

The interfacial protein plays a key role in stabilizing the emulsion by forming a physical barrier at the oil/water interface. Changes in the interfacial protein content can indicate the stability of the emulsion. Understanding the dynamics of protein adsorption at the interface helps to elucidate the gel formation mechanism. In order to better understand the formation process of the emulsion gel induced by HPP, the alteration in the content of protein in the water phase, which is the protein not adsorbed at the oil/water interface during the formation of the emulsion gel, was studied. The results are shown in Figure 6. During the HPP process, the unadsorbed protein content decreased with time. It can be seen from the figure that as the concentration of κ-CG increased, the proportion of unadsorbed proteins in the emulsion gel decreased, indicating that the addition of κ-CG facilitated the adsorption of WPI on the oil/water interface and its participation in the formation of the network structure. Cheng et al. also found that after adding 0.05% pectin, the interfacial protein content of the emulsion stabilized by zein particles increased significantly from 45% to 73%. This increase might be attributed to the fact that the pectin could promote particles to absorb onto the oil/water interface, preventing aggregation and coalescence by electrostatic repulsion and steric hindrance [18]. Therefore, the increase in the κ-CG concentration can increase the interfacial protein content in the gel network.

It can be seen that when the concentration of κ-CG was 0.8%, the proportion of unadsorbed protein gradually decreased with the increase in pressure, indicating that HPP was beneficial to the participation of protein in the network structure. When the κ-CG concentration was 1.0% and 1.2%, with the treatment pressure increased, the proportion of unadsorbed protein decreased first and then increased, reaching the lowest level at 500 MPa. This result is in agreement with the findings of Yang et al. [19], who studied the effect of HPP on the quality of protein adsorbed onto the interface of the emulsified layer. They found that HPP could significantly increase the total amount of protein adsorbed on the surface of fat particles, with the protein adsorption capacity showing a trend of first increasing and then decreasing with increasing pressure.

### 2.6. SDS-PAGE

**Figure 7:**
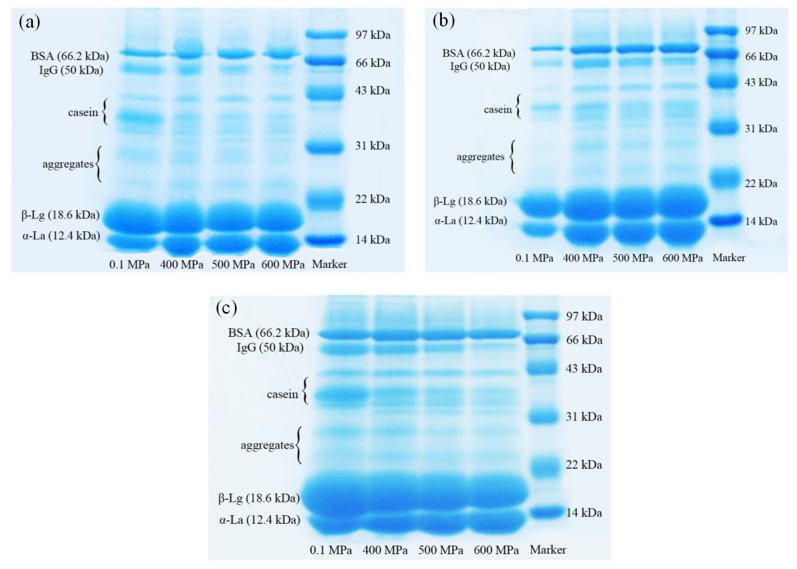
The electrophoretic band of unabsorbed proteins in emulsion gels under different pressure and κ-carrageenan (κ-CG) concentrations: (**a**) 0.8% κ-CG; (**b**) 1.0% κ-CG; (**c**) 1.2% κ-CG. Note: in Figure 7, the holding time is 30 min.

SDS-PAGE was performed on the water phase protein separated from the gel, and ImageJ software was used for grayscale analysis to quantify the changes in the content of each component represented by each band on each electrophoresis lane. The results are shown in Figure 7. As we all know, WPI includes β-lactoglobulin (β-Lg), α-lactalbumin (α-La), bovine serum albumin (BSA), immune globulin (IgG), lactoferrin, etc. Because the WPI used in this experiment was not pure, it can be seen from the figure that a small amount of casein was present in the lane. There were mainly six regions in one lane, representing α-La (12.4 kDa), β-Lg (18.6 kDa), dimers and trimers of α-La and β-Lg, casein, the heavy chain of IgG (50 kDa) and BSA (66.2 kDa) [20].

It can be seen from Table 2 that after HPP, the content of β-Lg, IgG and casein in the water phase of the gel significantly decreased, while the content of α-La and BSA increased significantly, indicating that HPP had a significant effect on the composition of the oil/water interface protein of the emulsion gel. After HPP treatment, the content of BSA and α-La of the interface protein decreased significantly, while the content of IgG, casein and β-Lg increased significantly (*p* < 0.05). When the κ-CG concentration was 0.8% and 1.0%, the content of IgG and casein at the oil/water interface significantly increased with the increase in pressure (400–600 MPa), while the content of α-La decreased significantly. When the concentration of κ-CG was increased to 1.2%, the change in the interface protein components with pressure was narrowed down. In addition, with the increase in pressure (400–600 MPa), there was generally no significant change in the content of β-Lg. With the increase in the κ-CG concentration, the proportion of casein in the interface protein increased, but the other components did not change significantly.

## 3. Conclusions

The addition of κ-CG significantly reduced the protein concentration required for the formation of emulsion gel induced by HPP and greatly reduced the swelling rate of the emulsion gel. The gel strength and storage stability of the composite emulsion gels increased with the increase in pressure (400–600 MPa) and holding time (10–30 min). When the pressure increased to 500 MPa, the stability of the curcumin in the emulsion gel significantly improved. When the ratio of WPI to κ-CG was 12:1 (κ-CG concentration is 1.0%), the photochemical stability and thermal stability of curcumin were higher than those of the other two ratios. Increasing the concentration of κ-CG had no significant effect on the T_2_ relaxation time of the gel, but the monolayer water and multilayer water were converted into immobilized water. The HPP significantly increased the mobility of monolayer water in the system, while the mobility of multilayer water and immobilized water was significantly reduced. Increasing the holding time and the concentration of κ-CG can both result in an increase in the interfacial protein content in the oil/water system, and the HPP treatment had a significant effect on the composition of the interfacial protein of the emulsion gel. After the HPP treatment, the content of bovine serum albumin and α-lactalbumin at the interface decreased significantly, while the content of immunoglobulin, casein and β-lactoglobulin increased significantly.

## 4. Materials and Methods

### 4.1. Materials 

WPI (purity > 95%) was purchased from Davisco Food International (Le Sueur, MN, USA). κ-CG (purity > 90%) was obtained from CP Kelco (Copenhagen, Denmark). Curcumin (purity > 97%) was acquired from China National Pharmaceutical Group Co., Ltd. (Shanghai, China). Medium chain triglycerides (MCTs, purity > 99%) were purchased from P.T. Musim Mas Group Ltd. (Singapore). The reagent test kit for measuring protein content was purchased from Nanjing Jiancheng Technology Co., Ltd. All reagents for electrophoresis were obtained from Beijing Solarbio Technology Co., Ltd. (Beijing, China). All other chemical agents were analytical grade and obtained from the Beijing Chemical Plant Co., Ltd. (Beijing, China). Water purified by a Milli-Q system (Millipore, MA, USA) was used for all the experiments.

### 4.2. Preparation of HPP-Induced WPI-CG Composite Emulsion Gel Loaded with Curcumin

#### 4.2.1. Preparation of WPI-CG Compound Solution

WPI solution (24% *w*/*v*) was prepared by weighing a certain mass of WPI powder, dissolving it in deionized water and then stirring with a magnetic stirrer at room temperature until WPI completely dissolved. A κ-CG solution (1.6%, 2.0%, 2.4% *w*/*v*) was prepared by dispersing a certain mass of powder in deionized water and stirring at room temperature until it was completely dissolved. After that, the WPI solution was mixed with the κ-CG solution in equal volumes. The WPI concentration in the final solution system was 12% (*w*/*v*), and the κ-CG concentrations were 0.8%, 1.0%, 1.2% (*w*/*v*), respectively. The compound solution was stirred overnight at room temperature and the pH of the system was adjusted to 7 using 1 mol/L NaOH. An amount of 0.02 wt% NaN_3_ was added as an antimicrobial agent.

#### 4.2.2. Preparation of Curcumin-Loaded Oil-in-Water Emulsion

Curcumin was dissolved in MCT on the basis of the method presented by Ma et al., with some modifications [21]. Briefly, 1 g curcumin was added to 200 g MCT, magnetically stirred for 10 min in the dark and then sonicated with an ultrasonic cell disruptor (405 W, 1 s interval) for 30 min. Then, the solution was centrifuged at 3000 rpm for 5 min to remove the undissolved powder. The supernatant was collected and stored at 25 °C in the dark for later use. A certain mass of curcumin-loaded MCT was added to the prepared WPI-CG compound solution to achieve an oil phase content of 30% (*w*/*w*). The system was sheared for 3 min at 10,000 rpm using a T25 high-speed disperser (IKA, Staufen, Germany) to form coarse emulsion, and then processed by a high-pressure homogenizer (NS1001L2K, Niro-Soavi, Parma, Italy) at 50 MPa for three times to form a fine emulsion.

#### 4.2.3. High Pressure Processing (HPP)

The prepared emulsion was cooled to room temperature and poured into a 50 mL plastic centrifuge tube, which was then put into a polyethylene bag and sealed using a vacuum packaging machine. HPP treatment was performed in a laboratory-scale high-pressure processor (L2-700/1, Tianjin Huatai Senmiao Biological Engineering Technology Ltd., Tianjin, China) with water as the transmitting medium. The treatment pressures in this experiment were 400 MPa, 500 MPa and 600 MPa, and the holding time was set to 10 min, 20 min and 30 min, respectively. The pressure rise rate was about 6.5 MPa/s, and the release rate was about 20 MPa/s. The treated samples were stored in a refrigerator at 4 °C for 24 h, and then the various parameters of the gels were measured.

### 4.3. Swelling Rate Measurement

The sample was cut into a cylinder with a height of 1 cm and a diameter of 1 cm, and the mass of the cylinder was accurately weighed and recorded as M_0_. The sample was then put into 20 mL of pH 7.0 deionized water and was swollen for 2 or 12 h. After absorbing the surface moisture, the sample was weighed and the mass was recorded as M_1_. The swelling rate of the sample was calculated by (M_1_ − M_0_)/M_0_.

### 4.4. Texture Profile Analysis (TPA)

Texture profile analysis was performed by using a TA-XT Plus Texture Analyser (Stable Micro System Co., Godalming, UK) at room temperature, according to the method described by Kotwaliwale, Bakane and Verma [22]. The cylindrical samples (18 mm in diameter, 20 mm height) were axially compressed with a cylindrical probe (P/6, stainless steel). The test speed was kept constant at 100 mm/min, the compression deformation was 50% of the sample, and the trigger force was 0.375 N, while the force range was 250 N. The TPA parameters, namely hardness (N), springiness (dimensionless), cohesiveness (dimensionless) and chewiness (N), were computed.

### 4.5. Stability of Curcumin in the Emulsion Gel

#### 4.5.1. Photochemical Stability

The photochemical stability of curcumin in the emulsion gels was assessed using a light cabinet (Q-Sun, Q-Lab Corporation, Westlake, OH, USA). All samples were kept under light conditions (35 °C, 0.35 W/m^2^) for 4 h, and the curcumin content in the emulsion gel was collected at intervals of 1 h. The retained content of curcumin was determined as described by Dai et al., with some modification [23]. Briefly, about 0.1 g of emulsion gel was weighed, and the mass was recorded. Then, it was mashed with a glass rod and put into 5 mL 80% (*v*/*v*) ethanol solution. The suspension was ultrasonically extracted for 5 min (300 w), shaken with a vortex oscillator for 1 min (1500 rpm) and centrifuged for 5 min (10,000 rpm, 25 °C). The supernatants of three times of centrifugation were collected together. The absorbance of the samples at 426 nm was determined using a UV1800 spectrophotometer (Shimadzu Corporation, Kyoto, Japan). All experiments were performed in triplicate. Curcumin content in the emulsion gel was calculated from the standard curve.

#### 4.5.2. Thermal Stability

Freshly prepared samples were treated at 80 °C for 4 h in a water bath kept in the dark, and the samples were collected at regular intervals of 1 h. The curcumin retained in these samples was extracted and determined as described in the part mentioned above. All experiments were performed in triplicate.

### 4.6. Low-Field Nuclear Magnetic Resonance (LF-NMR)

The gel samples were cut into cylinders of the same shape and size with a height of less than 4 cm, placed in a glass tube (40 mm in diameter) and inserted into the NMR probe of a Pulsed NMR analyzer (VTMR20-010V-I, Suzhou Niumag Analytical Instrument Co., Ltd., Suzhou, China). The analyzer was operated at a resonance frequency of 21 MHz at room temperature. Spin–spin relaxation times of ^1^H (T_2_) were measured using the Carr–Purcell–Meiboom–Gill (CPMG) sequence. T_2_ was measured, yielding a τ-value of 300 μs. Data from 10,000 echoes were acquired as 4 scan repetitions. The repetition time between subsequent scans was 3000 ms. Post-processing of NMR T_2_ data, including distributed exponential fitting of CPMG decay curves, was performed by Multi-Exp Inv Analysis software version 2.0 (Niumag PQ001, Suzhou Niumag Analytical Instrument Co., Ltd., Suzhou, China). Four relaxation times (T_21_, T_22_, T_23_ and T_24_) and their corresponding water populations (P_21_, P_22_, P_23_ and P_24_) were recorded.

### 4.7. The Contents of Interface Protein

The prepared emulsion gel was centrifuged at 15,000× *g* for 90 min, and the water at the bottom of the centrifuge tube was carefully sucked with a syringe. The gel was then centrifuged for 30 min twice, and the water extracted from the three times of centrifugation was collected and combined. The extracted aqueous solution was diluted to 5 mL with 10 mM pH 7 phosphate buffer solution (PBS), mixed and passed through 0.45 μm and 0.22 μm filters in sequence.

The content of protein was determined by the Coomassie Brilliant Blue Method using the Total Protein determination kit (Nanjing Jiancheng Technology Co., Ltd., Nanjing, China, #A045-2) according to the protocol of the kit. This experiment indirectly analyzed the content of interface protein in the emulsion gel through measuring the content of unadsorbed protein in the aqueous solution.

### 4.8. Sodium Dodecyl Sulfate-Polyacrylamide Gel Electrophoresis (SDS-PAGE)

The protein solution obtained from Section 3 was subjected to sodium dodecyl sulfate-polyacrylamide gel electrophoresis (SDS-PAGE) analysis. The SDS-PAGE analysis was performed using a vertical gel electrophoresis system (BG-Power300, Beijing BayGene Biotechnology Co., Ltd., Beijing, China). The PAGE gels were prepared using the Polyacrylamide Gel Preparation kit (Beijing Solarbio Technology Co., Ltd., #P1200). The stacking gel was 10% (*w*/*v*) acrylamide and the separation gel was 12% (*w*/*v*) acrylamide. Samples were mixed 3:1 with 4× Laemmli sample buffer (with DTT) (Solarbio, #P1015) before loading. The running buffer consisted of 0.6% (*w*/*v*) Tris, 2.88% (*w*/*v*) glycine and 0.1% (*w*/*v*) SDS. After electrophoresis, the gels were stained in Coomassie Brilliant Blue Quick Staining Solution (Solarbio, #P1300) and then destained according to the protocol. The gels were photographed with an AI600RGB gel imager (General Electric Company, Atlanta, GA, USA) and then processed with ImageJ2 software (National Institutes of Health, Stapleton, NY, USA).

### 4.9. Statistical Analysis

All samples were measured in triplicate. Data were analyzed using the software package SPSS 22.0 (SPSS Inc., Chicago, IL, USA). Results were reported as the mean value and standard deviation (SD). Statistical differences were determined by one-way analysis of variance (ANOVA) with the Duncan procedure, and differences in the main effects were identified to be significant with *p* < 0.05.

## Figures and Tables

**Table 1:** LF-NMR spin–spin relaxation time (T_2_) and peak proportion (P) of emulsion gels.

Presssure	κ-CG Concentration	T_2_ (ms)				P (%)			
		T_21_	T_22_	T_23_	T_24_	P_21_	P_22_	P_23_	P_24_
0.1 MPa	0.8%	2.57 ± 0.53 ^a^	35.38 ± 2.83 ^a^	238.73 ± 34.61 ^a^	/	1.83 ± 0.06 ^b^	3.33 ± 0.25 ^b^	90.67 ± 0.31 ^a^	/
	1.0%	2.21 ± 0.17 ^a^	37.65 ± 2.97 ^a^	231.01 ± 10.00 ^a^	/	1.87 ± 0.06 ^ab^	3.57 ± 0.21 ^b^	90.53 ± 0.32 ^a^	/
	1.2%	2.11 ± 0.25 ^a^	37.65 ± 3.56 ^a^	231.01 ± 2.34 ^a^	/	2.17 ± 0.25 ^a^	3.67 ± 0.21 ^a^	90.47 ± 0.25 ^b^	/
400 MPa	0.8%	3.69 ± 0.30 ^a^	24.77 ± 0.90 ^a^	174.75 ± 1.56 ^a^	/	1.77 ± 0.15 ^a^	2.4 ± 0.01 ^a^	93.4 ± 0.75 ^a^	0.00 ^c^
500 MPa		3.25 ± 0.71 ^a^	21.54 ± 0.20 ^b^	174.75 ± 11.00 ^a^	1355.93 ± 96.59 ^a^	1.60 ± 0.10 ^a^	1.97 ± 0.15 ^b^	94.07 ± 0.45 ^a^	0.27 ± 0.06 ^b^
600 MPa		3.05 ± 0.24 ^a^	19.67 ± 1.62 ^b^	151.99 ± 0.67 ^b^	1072.27 ± 58.45 ^b^	1.50 ± 0.20 ^a^	1.57 ± 0.32 ^b^	93.4 ± 0.10 ^a^	1.03 ± 0.15 ^a^
400 MPa	1.0%	2.73 ± 0.75 ^a^	24.77 ± 0.90 ^a^	174.75 ± 5.64 ^a^	/	1.60 ± 0.17 ^a^	1.87 ± 0.12 ^a^	94.33 ± 0.91 ^a^	0.00 ^c^
500 MPa		2.33 ± 0.32 ^a^	18.74 ± 0.35 ^b^	151.99 ± 2.46 ^b^	1072.27 ± 56.46 ^b^	1.60 ± 0.10 ^a^	1.73 ± 0.06 ^a^	93.33 ± 0.40 ^a^	0.80 ± 0.00 ^b^
600 MPa		2.53 ± 0.74 ^a^	17.52 ± 1.73 ^b^	151.99 ± 2.35 ^b^	1232.85 ± 89.45 ^a^	1.20 ± 0.57 ^a^	1.57 ± 0.07 ^b^	93.45 ± 0.35 ^a^	1.43 ± 0.06 ^a^
400 MPa	1.2%	2.83 ± 0.62 ^a^	21.54 ± 0.65 ^a^	174.75 ± 1.65 ^a^	1629.75 ± 62.32 ^a^	1.87 ± 0.12 ^a^	1.87 ± 0.15 ^a^	93.70 ± 0.20 ^a^	0.27 ± 0.06 ^c^
500 MPa		2.16 ± 0.58 ^a^	17.92 ± 1.41 ^b^	151.99 ± 5.13 ^b^	1072.27 ± 98.65 ^c^	1.57 ± 0.06 ^b^	1.67 ± 0.06 ^b^	93.97 ± 0.29 ^a^	1.03 ± 0.06 ^b^
600 MPa		2.14 ± 0.47 ^a^	17.11 ± 1.41 ^b^	151.99 ± 2.24 ^b^	1232.85 ± 20.20 ^b^	1.27 ± 0.06 ^c^	1.43 ± 0.06 ^c^	93.73 ± 0.64 ^a^	1.43 ± 0.06 ^a^

Note: Different letters in the same column indicate a significant difference (*p* < 0.05, n = 3). The holding time is 30 min.

**Table 2:** The percentage of the components represented by each band in the SDS-PAGE image.

Pressure (MPa)	κ-CG Concentration	The Percentage of Components in WPI (%)				
		BSA	IgG	Casein	β-Lg	α-La
0.1	0.8%	10.42 ± 0.25 ^c^	8.72 ± 0.07 ^a^	15.13 ± 0.14 ^a^	50.98 ± 0.24 ^a^	14.74 ± 0.30 ^d^
400		12.43 ± 0.33 ^ab^	7.20 ± 0.56 ^b^	13.36 ± 0.37 ^b^	43.84 ± 0.32 ^b^	23.39 ± 0.58 ^c^
500		12.79 ± 0.39 ^a^	5.89 ± 0.33 ^c^	11.58 ± 0.35 ^c^	43.46 ± 0.43 ^b^	26.40 ± 0.42 ^b^
600		11.94 ± 0.06 ^b^	4.00 ± 0.39 ^d^	10.02 ± 0.28 ^d^	43.64 ± 0.27 ^b^	30.41 ± 0.42 ^a^
0.1	1.0%	7.27 ± 0.11 ^c^	7.51 ± 0.06 ^a^	14.41 ± 0.13 ^a^	51.66 ± 0.32 ^a^	19.16 ± 0.12 ^d^
400		10.04 ± 0.04 ^b^	6.48 ± 0.21 ^b^	11.03 ± 0.25 ^b^	45.97 ± 0.22 ^bc^	26.48 ± 0.26 ^c^
500		9.78 ± 0.26 ^b^	4.81 ± 0.18 ^c^	9.47 ± 0.23 ^c^	45.50 ± 0.35 ^c^	30.45 ± 0.32 ^b^
600		10.41 ± 0.14 ^a^	3.38 ± 0.36 ^d^	8.87 ± 0.09 ^d^	46.15 ± 0.02 ^b^	31.19 ± 0.42 ^a^
0.1	1.2%	7.84 ± 0.35 ^c^	6.28 ± 0.29 ^b^	8.42 ± 0.38 ^c^	51.43 ± 0.58 ^a^	26.03 ± 0.45 ^c^
400		11.18 ± 0.24 ^b^	7.62 ± 0.15 ^a^	9.92 ± 0.57 ^a^	43.57 ± 0.31 ^b^	27.71 ± 0.54 ^b^
500		11.73 ± 0.20 ^a^	6.35 ± 0.12 ^b^	8.97 ± 0.31 ^bc^	42.74 ± 0.52 ^b^	30.21 ± 0.52 ^a^
600		11.46 ± 0.21 ^ab^	4.86 ± 0.29 ^c^	9.53 ± 0.51 ^ab^	43.41 ± 0.35 ^b^	30.75 ± 0.37 ^a^

Note: Different letters in the same column indicate a significant difference (*p* < 0.05, n = 3). The holding time is 30 min.

## Data Availability

All data generated or analyzed during this study are included in this published article.
